# Cross-protection between *Trypanosoma congolense* strains of low and high virulence

**DOI:** 10.1016/j.vetpar.2009.04.006

**Published:** 2009-07-07

**Authors:** J. Masumu, T. Marcotty, S. Geerts, J. Vercruysse, P. Van den Bossche

**Affiliations:** a University of Pretoria, Department of Veterinary Tropical Diseases, Onderstepoort, South Africa; b Institute of Tropical Medicine, Animal Health Department, Nationalestraat 155, B-2000 Antwerp, Belgium; c Ghent University, Vakgroep Virologie, parasitologie en Immunologie, Salisburylaan 133, B-9820 Merelbeke, Belgium

**Keywords:** *Trypanosoma congolense*, Cross-protection, Virulence, Mouse, Zambia

## Abstract

The aim of this study was to assess the existence of possible cross-protection between *Trypanosoma congolense* strains of low and extreme virulence circulating in the same trypanosomiasis focus. Groups of six mice were infected using one of three strains of low virulence and challenged with one of three strains of extreme virulence. A group of six mice was used as control for each strain of low and extreme virulence. The results showed that mice infected with one of the strains of extreme virulence developed high parasitaemia and a significant drop of the PCV compared to mice infected with a strain of low virulence and challenged with one of the strains of extreme virulence. With an exception of one strain of extreme virulence (strain F), the survival time of mice infected with the strains of extreme virulence was shorter compared to mice infected with strains of low virulence and subsequently challenged with a strain of extreme virulence. These results suggest that in an area where trypanosomes of various virulence profiles circulate, livestock infected with *T. congolense* strains of low virulence can be protected against the adverse effects of extremely virulent *T. congolense* strains.

## 1 Introduction

Trypanosomiasis is an important constraint to livestock and rural development in extensive areas of sub-Saharan Africa. The disease is caused by various pathogenic trypanosome species but its impact on production varies substantially between trypanosome and livestock species. *Trypanosoma congolense* is considered as an important pathogenic trypanosome species affecting livestock but its effect on the health of susceptible livestock varies substantially between strains (Masumu et al., 2006a). Some strains, the highly virulent ones, cause an acute disease with high mortality whereas other strains (with low virulence) result in a chronic mild infection. Since susceptible livestock in a tsetse-infested area is challenged continuously, it thus seems that strain composition of the *T. congolense* population will determine the impact of the disease in a particular area.

In a study conducted in a bovine trypanosomiasis endemic area of eastern Zambia, almost 20% of the *T. congolense* strains circulating in the cattle population were highly pathogenic whereas the remaining trypanosome strains (80%) were of moderate or low virulence (Masumu et al., 2006a). Notwithstanding this proportion of extremely virulent strains and the low trypanocidal drug treatment frequency (Van den Bossche et al., 2000), the disease in livestock in the area has an endemic character (Doran and Van den Bossche, 1999). This suggests possible interaction between circulating trypanosome strains.

It is well known that humoral immunity can develop as a result of homologous trypanosome challenge (Wellde et al., 1981; Nantulya et al., 1984). Such immunity is based on the production of specific neutralizing antibodies by the host that react with homologous but not heterologous trypanosome strains. The degree of protection is related to the length of infection with greater protection being observed after chronic infections sometimes resulting in self-cure (Wellde et al., 1981; Nantulya et al., 1984). Besides humoral immunity, cross-protection in the development of a heterologous trypanosome strain has also been described. Here protection requires the presence of an active infection and antibodies were not shown to be implicated in the process (Morrison et al., 1982; Luckins and Gray, 1983). Although the cross-protection between trypanosome strains has been described by several researchers, little attention has been paid to the practical implications of this phenomenon in a geographical limited area and how this phenomenon can contribute to the establishment of a trypanosomiasis endemic situation in a susceptible livestock population even in the presence of virulent trypanosome strains.

To investigate the role of cross-protection between trypanosome strains on the expression of the virulence of circulating trypanosome strains and the persistence of virulent strains in a susceptible host population, the cross-protection between strains of various levels of virulence isolated from cattle in a trypanosomiasis endemic area of eastern Zambia was investigated.

## 2 Material and methods

### 2.1 Trypanosome strains

Six *T. congolense* strains (Savannah subgroup) belonging to the same trypanosome population and isolated from cattle in Katete District of eastern Zambia were used in the experiments. These isolates were cloned in mice and have been shown to exhibit different genetic profiles when characterized using a modified amplified fragments length polymorphism (AFLP) technique (Masumu et al., 2006b). Three strains (A, B and C), belonging to the low virulence category (LVS) with median survival times of >30 days in mice, were selected randomly from a total of 12 strains. The three other strains (D, E and F) were extremely virulent (EVS) with a short prepatent period, a high parasitaemia, and a short median survival time (between 5 and 9 days) in mice. They were selected randomly from a total of six strains. The virulence profiles of these strains and the study area where these isolates were collected were described elsewhere (Masumu et al., 2006a).

### 2.2 Experimental design

A total of 12 groups of six mice (OF1) were infected intraperitoneally with approximately 10^5^ trypanosomes LVS in 0.2 ml of Phosphate buffered Saline Glucose (PSG). Each of the 3 LVS was used in 4 different groups (Table 1). To ensure that the infection had established, the parasitaemia of injected mice was checked three times a week by direct examination of tail blood. Twenty days after the injection of the LVS, the nine of the 12 groups plus 3 groups of 6 non-infected mice were challenged with one of the EVS. This was done in such a way to have one group for each LVS and EVS combination, including controls (Table 1). The EVS challenge was conducted as described above for the LVS infection. For all mice, the level of parasitaemia, the mortality and the development of anaemia were recorded. The parasitaemia was estimated daily during the first two weeks and thereafter every two days using wet tail-blood film method (Herbert and Lumsden, 1976). A mouse was considered parasitologically negative when no trypanosomes were detected in at least 50 microscopic fields. Mortality was recorded daily. The Packed Cell Volume (PCV) of tail-blood was measured using the micro-centrifugation method. It was measured before infection, every two days during the first two weeks after infection, and once a week for the remainder of the experiment (up to 60 days post-infection). Animal ethics approval for the experimental infections was obtained from the Ethics Commission of the Institute of Tropical Medicine, Antwerp, Belgium (Ref DG001-PD-M-TTT).

### 2.3 Genetic analysis

To determine whether trypanosomes of extreme virulence developed in mice infected with a strain of low virulence, the genetic profiles of the DNA of trypanosome isolated from the blood before and after challenge were compared with the profiles of the EVS and the LVS. For this purpose, blood was collected from the infected mice and trypanosome pellets were produced using the mini column technique (Lanham and Godfrey, 1970). After extraction of the DNA, the genetic analysis was performed using a modified amplified fragment length polymorphism (Masumu et al., 2006b) and the profiles were compared.

### 2.4 Statistical analysis

The statistical analyses were carried out using Stata 9.0 software (StataCorp., 2006). The PCV values measured on surviving mice on days 0 and 7 post EVS challenge (20 and 27 days after the LVS infections, respectively) were analysed in a cross-sectional linear regression model. To ensure normality of the response variable, the square root of the PCV values were arcsin transformed (Osborne, 2002). The virulence category, the day post-challenge and the interaction between them were used as categorical explanatory variables. Subgroups resulting from the combination of the strains used for primo-infection and for challenge were considered as clusters. Individual mice, within a group of six were considered as a second cluster level. Therefore, a three-level adaptive generalized linear latent and mixed model (GLLAMM) was applied on the PCV data using individual mice nested in subgroups as random effects. In a second GLLAMM, the survival time of mice from the three different scenarios (primo infection with or without challenge and challenge without primo infection) was used as response variable. Whether the mice were primo-infected or not and challenged or not was taken as explanatory variable. Random effects were the combinations of strains as described above. The dataset was transformed to apply a Poisson regression as an approximation of a Cox proportional hazard ratio (http://www.bepress.com/ucbbiostat/paper160). For all groups, including the LVS control groups, the time origin was the day of EVS challenge, 20 days after the LVS primo-infection of mice.

## 3 Results

All the mice that were inoculated with one of the three LVS developed a parasitaemia. At the moment of challenge with one of the EVS (20 days after LVS infection), the parasitaemia ranged between 10^7.2^ and 10^8.1^ trypanosomes/ml of blood. Challenge with an EVS and the subsequent mixed infection did not result in a substantial rise in parasitaemia, unlike the control mice infected with one of the EVS, which showed high parasitaemia (data not shown). Genetic analysis of trypanosomes collected seven days after challenge with one of the EVS revealed the presence of mixed (LVS and EVS) infections.

**Fig. 1:**
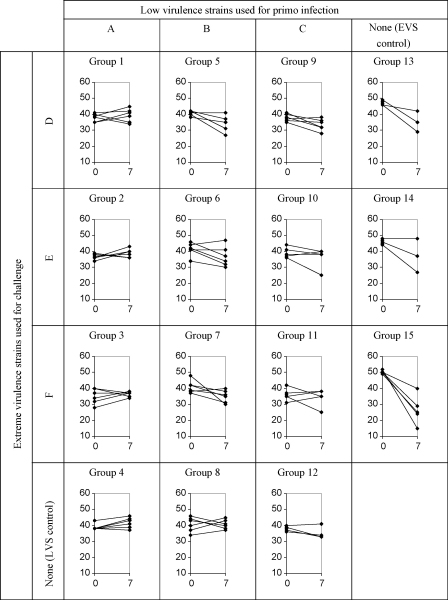
PCV values in mice (*n* = 6) infected with *Trypanosoma congolense* strains of low virulence (A, B or C) and challenged with one of three extremely virulent *T. congolense* strains (D, E or F) on day 0 and 7 post-challenge. Ordinate: PCV (%). Abscissa: day post-challenge (the origin, day 0, is 20 days after the primo-infection of mice with low virulence strains, including for the LVS controls). On day 7 post-challenge, the decline in the average PCV of the control EVS groups was significantly greater (*p* < 0.001) than in the primo-infected groups. The PCV of the LVS control groups did not differ significantly from that of the challenged groups (*p* = 0.10).

During the first 7 days post-challenge, the decline in the average PCV of mice infected with one of the EVS (on average 16.8%, from 47.8% to 31.0%) was significantly greater (*p* < 0.001) than in mice previously infected with a LVS (on average 2.4%, from 38.5% to 36.1%) (Fig. 1). Moreover, the PCV of control mice infected with one of the LVS did not differ significantly (*p* = 0.10) from that of mice infected with a LVS and challenged with one of the EVS.

**Fig. 2:**
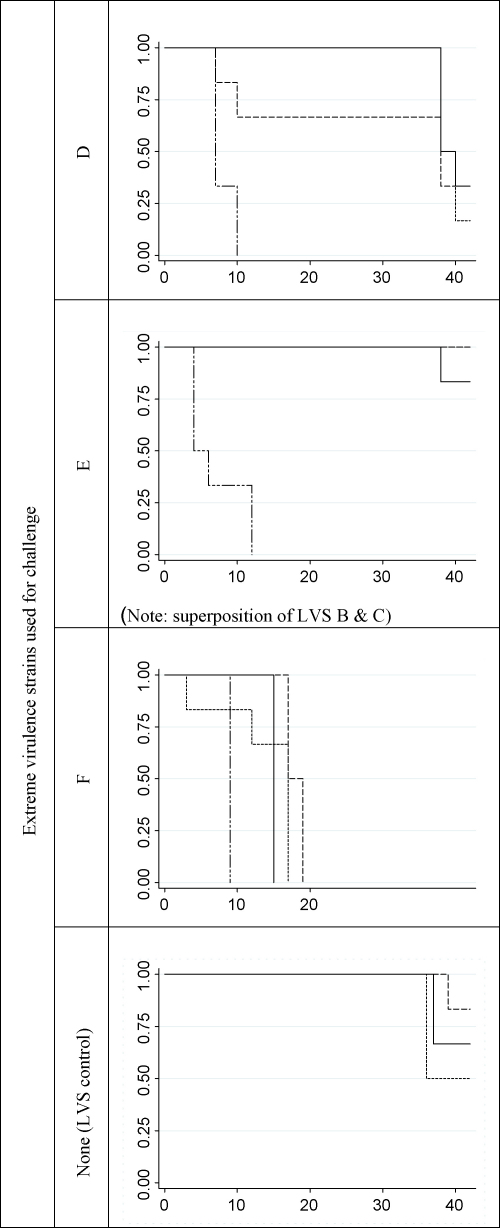
Kaplan-Meier graphs illustrating the survival of mice in the different groups: mice infected with LVS A (———), B (- - - - -), C (………) or none (– ·· – ·· –). Ordinate: proportion of surviving mice. Abscissa: days post-challenge (the origin, day 0, is 20 days after the primo-infection of mice with low virulence strains, including for the LVS controls). The survival in the EVS control groups was significantly shorter than in the LVS control group (relative risk of dying was 333; *p* < 0.001) and in the primo-infected mice (relative risk of dying was 89; *p* < 0.001).

Mice infected with one of the EVS had a significantly higher relative risk of dying than those infected with a LVS (relative risk of dying was 333; *p* < 0.001) or mice infected with a LVS and subsequently challenged with an EVS (relative risk of dying was 89; *p* < 0.001). However, the survival time of mice infected with a LVS and challenged with two of the EVS (strains D and E) was longer compared to that of mice challenged with the strain F (all mice died before day 19). These results are shown in Fig. 2.

## 4 Discussion

The data presented in these studies revealed that challenge of animals infected with a trypanosome strain of low virulence, and subsequently challenged with an extremely virulent heterologous strain, did not prevent the establishment of this strain and its development as a mixed *T. congolense* infection. However, whereas previously protection was assessed at the level of the trypanosome's development (Morrison et al., 1982; Luckins and Gray, 1983), our results suggest that protection may also be present at the level of the parasite's expression of its virulence. Indeed, despite the establishment of a secondary infection with a strain of extreme virulence, the virulent strain's parasitaemia, its effect on the PCV and the mortality rate of infected animals were significantly reduced in the experimental animals previously infected with a strain of low virulence. This was certainly so for challenge with two of the strains of extreme virulence (strains D and E) and to a lesser extent after challenge with strain F.

Although the results from these experiments cannot be extrapolated to strains from other areas, they show that under the circumstances prevailing in eastern Zambia and between the trypanosome strains randomly selected for the experiments, an infection with a *T. congolense* strain with low virulence may confer protection against the adverse effects of a secondary infection with an extremely virulent *T. congolense* strain. This beneficial effect of coexistence of parasites with different level of virulence and circulating in a particular area has previously been described in malaria where an infection with *Plasmodium vivax* attenuates the negative effect of an infection with *P. falciparum* (Luxemburger et al., 1997). Moreover, a chronic *P. falciparum* infection appears to offer cross-protection against heterologous challenge with the same parasite species (Smith et al., 1999).

In a previous study, it was observed that the impact of trypanosomiasis occurring in a particular area may be related to the virulence of the circulating trypanosome strains (Masumu et al., 2006a). In the assumption that the results obtained in both studies in mice can be extrapolated to cattle, it can be hypothesized that the impact of the disease in a particular area will likely be related, not only to the overall virulence profile of trypanosome strains circulating in the area, but also to protection against trypanosomes of extreme virulence that is conferred by previous infections by trypanosomes of low virulence. Consequently in areas where low virulent trypanosome strains induce protection against most present trypanosomes of extreme virulence (case of strains D and E) the disease will have a low impact on animal health and livestock production. On the other hand, in areas where a low cross-protection is observed between most trypanosomes of extreme and low virulence circulating in livestock (case of strain F) the disease will likely have a high impact on animal health and production.

In terms of disease control (Snow and Rawlings, 1999), it is therefore speculated, as for malaria (Böete and Paul, 2006), that control strategies that disproportionally negatively impact on trypanosomes of low virulence should be avoided. A disruption of the interaction between trypanosomes of extreme and low virulence circulating in a particular area may result in an increased disease burden on animal health and consequently on livestock production. This could be the case when, for example, trypanocidal drugs are used indiscriminately on sick and healthy animals. Results from our study suggest that curative treatment of sick animals is more appropriate than chemoprophylaxis or blanket chemotherapy to mitigate the burden of trypanosomiasis endemic situations. Further studies are needed to verify these hypotheses.

## Figures and Tables

**Table 1:** Groups of mice (*n* = 6) infected with *Trypanosoma congolense* strains of low virulence (A, B or C) and challenged with one of three extremely virulent *T. congolense* strains (D, E or F) 20 days later (groups 4, 8 and 12 were used as control for the low virulence strains while groups 13–15 were used as control for the extremely virulent strains).

Group	LVS infections (day −20)	EVS infections (day 0)
1	A	D
2	A	E
3	A	F
4	A	

5	B	D
6	B	E
7	B	F
8	B	

9	C	D
10	C	E
11	C	F
12	C	

13		D
14		E
15		F
